# Thermafil: A New Clinical Approach Due to New Dimensional Evaluations

**DOI:** 10.2174/1874210601812010173

**Published:** 2018-02-22

**Authors:** G. Vittoria, G. Pantaleo, A. Blasi, G. Spagnuolo, A. Iandolo, M. Amato

**Affiliations:** 1 Department of Neurosciences, Reproductive and Odontostomatological Sciences, University of Naples Federico II, Naples, Italy; 2 Department of Medicine and Surgery, University of Salerno, Salerno, Italy

**Keywords:** Thermafil, Stereo microscope, Endodontic obturation, Gutta-percha, Zetaline stereo evolution, NiTi rotary system

## Abstract

**Background:**

There are a lot of techniques to obturate the root canals, but lateral condensation of gutta-percha is the most used one. An important aspect of thermafil is the error margin tolerated by the manufacturer in the production of plastic carriers. In literature, there is no evidence about discrepancy percentage between different carriers. It is demonstrated that the error margin of gutta-percha is 0.5% and is 0.2% for metal files (ISO standards).

**Objective:**

The aim of this study was to evaluate the real dimensions of thermafil plastic carriers observed by the stereo microscope measuring the dimensional discrepancy between them.

**Methods:**

For this study, 80 new thermafil (Dentsply Maillefer) have been selected. 40 thermafil 0.25 and 40 thermafil 0.30. Through 60X stereo microscope, the dimensions of the plastic carrier tips have been measured. The dimensions of the plastic carrier were also measured after a heating cycle. ZL GAL 11TUSM (Zetaline stereo evolution) microscope was used to observe the samples. Measurements were made through a dedicated software (Image Focus). All samples were analysed at 60X.

**Results:**

A non-parametric paired test (Wilcoxon test) was used to compare baseline and after heating values; *p*-values ≤ 0.05 were assumed to be statistically significant.

**Conclusion:**

The samples we measured showed a mean value of the diameters in Thermafil 25 that was 0.27 mm, for Thermafil 30 the mean value was 0.33 mm.

We have measured a dimensional variable of 8% in the 25 group while in group 30 the maximum possible variation found was 4%, that’s why we propose a new protocol of obturation with thermafil. We can also conclude that a single heating process does not affect clinically the plastic carrier dimensions.

## 1 INTRODUCTION

Many techniques have been developed to obturate the root canals, but lateral condensation of gutta-percha has been the widely used technique [1-3]. Recently, the manufacturers have introduced their individual tapered gutta-percha master cones to match the taper and apical sizes of the canals prepared with the respective NiTi rotary system claiming that the matched taper points can fill tapered canals effectively since they correspond to canal shapes created by instruments of similar taper [4]. Manufacturers also recommend obturation of the root canals prepared with NiTi rotary systems by using their respective thermoplasticized gutta-percha coated carrier systems.

Obturators are designed to correspond to the ISO standard file sizes and variable tapered NiTi rotary files. It was stated that these techniques can produce a homogenous mass in the root canal with a better gutta-percha-to-sealer ratio than that achieved with cold lateral condensation [5, 6].

Thermafil technique was introduced by Ben Johnson in 1978 and in the early 1990s it was introduced into the market as a K-File covered by gutta-percha that was inserted into the canal after bunsen flame heating [7].

Thermafil is a gold standard technique in case of severe curves, double curves, narrow canals and long canals [2, 8, 9]

The current configuration of Thermafil includes a central radiopaque plastic core (carrier) surrounded by a gutta-percha layer that will be heated in an electric oven to ensure thermoplasticization.

Thermafil obturators are available in 17 sizes, from 0.20 to 1.40 mm of tip diameter, with carrier taper of about 4%. Thermafil gutta-percha covers the carrier for about 16 mm [10]. For the selection of the correct obturator are available Ni-Ti verifiers with a diameter of 0.20 to 0.90 mm [11].

Some authors [12, 13] propose to use Thermafil plastic carrier from which the gutta percha has been removed (denudate carrier) instead of Ni-Ti verifiers, as the carrier reproduces the conditions occurring during filling operations better than the Ni-Ti verifier, so it is suggested to clinicians to denudate a series of carriers that are used only as “verifiers”, and then choose a corresponding obturator to seal the canal.

Pasqualini *et al*. [14] demonstrated *in vitro* that there is less apical infiltration selecting the denuded carrier reaching 0.5 mm from the apex that engage against the canal walls instead of selecting an obturator referring only to the correspondence between the apical final shaping diameter and the obturator size.

Another important aspect of thermafil is the error margin tolerated by the manufacturer in the production of plastic carriers. In literature, there is no evidence about discrepancy percentage between the different carriers.

In the literature, it is demonstrated that the error margin of guttapercha is 0.5% and is 0.2% for metal files (ISO standards) [8, 15].

The aim of this study was to evaluate the real thermafil plastic carriers dimensions observed by the stereo microscope measuring the dimensional discrepancy between them.

## 2 MATERIALS AND METHODS

A sample of 80 new thermafil (Dentsply Maillefer) was selected, 40 with 0.25 diameter and 40 with 0.30 diameter. Four groups were created for the study: Group 1 composed by 40 plastic carriers with 0.25 diameter; Group 2 composed by 40 plastic carriers with 0.30 diameter; Group 3 composed by 20 preheated 0.25 plastic carriers; Group 4 composed by 20 preheated 0.30 plastic carriers.

In all groups, thermafil has been denudated by gutta-percha by a single operator, using fingers and making sure not to apply strong pressures. No sharp tools were used to avoid deformation of plastic carriers. After measurements, the carriers of group 3 and 4 were covered again with gutta-percha and heated using the thermaprep plus oven (Dentsply Maillefer) set to 20-25 for group 3 (almost 20 seconds heating) and set to 30-60 for group 4 (almost 41 seconds heating). After 30 minutes of cooling carriers were denudated again by the same operator.

**Fig. (1):**
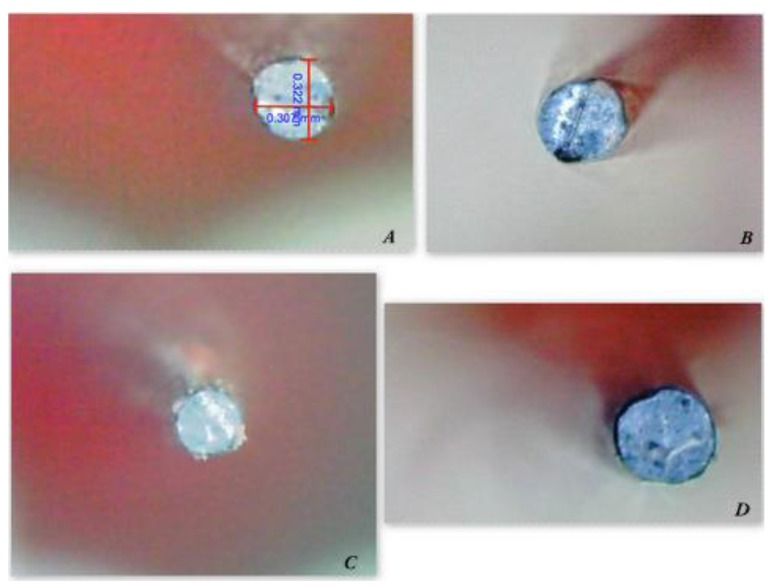
A - 60X image of a plastic carrier tip with 2 measuring axes; B - 60X image of a plastic carrier tip; C - 60X image of a plastic carrier tip with gutta-percha residue removed later; D - 60X image of a plastic carrier tip showing its irregularity.

Before measurements of all plasticized carriers, they were observed at 60X to evaluate the presence of guttapercha traces. Any trace has been removed before proceeding to measurements (Fig. **1**).

ZL GAL 11TUSM (Zetaline stereo evolution) microscope was used for observation of the samples. Measurements were made through a dedicated software (Image Focus). All samples were analysed at 60X. Samples were all placed in the same position before microscope analysis.

Measurements were taken only when all the plastic carrier tip surface was focused. As the plastic carrier tips were not perfectly circular, two measurements were taken for each sample, horizontal and vertical diameter.

To calculate the dimensional variability, we needed a reference value of the plastic core dimension. In literature or in the manufacturer’s indications this value is missing (the size of plastic core of a thermafil 25 and the size of the plastic core of thermafil 30). That’s why we compared our measurements not only with our samples media values, but also with an ideal value deduced by 2 literature statements:

- Gutta-percha exceeds the carrier of about 1mm
- Thermafil has a conicity of about 4%.

The ideal reference values which have been arbitrarily attributed, will therefore be 0.29 for Thermafil 25 and 0.34 for thermafil 30.

### 2.1 Statistical Analysis

First of all the two ideal reference values of diameters (0,29 mm and 0,34 mm) were transformed in areas: for each group an ideal area has been calculated, assuming a perfectly circular shape. A tolerance of 0.02 mm for the diameters has been accepted, the ideal areas were 0.066 ± 0.009 mm^2^ for Group 1 and 0.091 ± 0.011 mm^2^ for Group 2.

Then the tip area for each single plastic carrier has been calculated using the following formula for the ellipse area: (vertical axis / 2 x horizontal axis / 2 xπ) and the results were compared with the ideal values. The same was repeated for carriers after heating.

A non-parametric paired test (Wilcoxon test) was used to compare baseline and after heating values; *p*-values ≤ 0.05 was assumed to be statistically significant.

## 3 RESULTS

In Group 1, only 11 samples showed an area within the ideal range (metal tolerance), while 27 had a smaller area and 2 a larger one.

In Group 2, 34 samples had correspondence to the ideal range while 6 had a smaller area; no sample showed a wider area than the ideal one.

After heating, the areas were statistically wider than baseline in both groups (*p* = 0.0001 and *p* = 0.002 respectively), but this variation did not significantly affect the clinical appearance. So, in Group 1, only a sample after heating increased its area to fit the ideal parameters, while no variations in group 2 were reported.

The mean value of the diameters in Group 1 was 0.27 mm with a standard deviation of 0.02 (0.01); in group 2 the mean value was 0.33mm with a standard deviation of 0.01; in group 3 the mean value was 0.27 mm (0.26) with a standard deviation of 0.01 (0.02); in group 4 the mean value was 0.33mm with a standard deviation of 0.01.

**Fig. (2):**
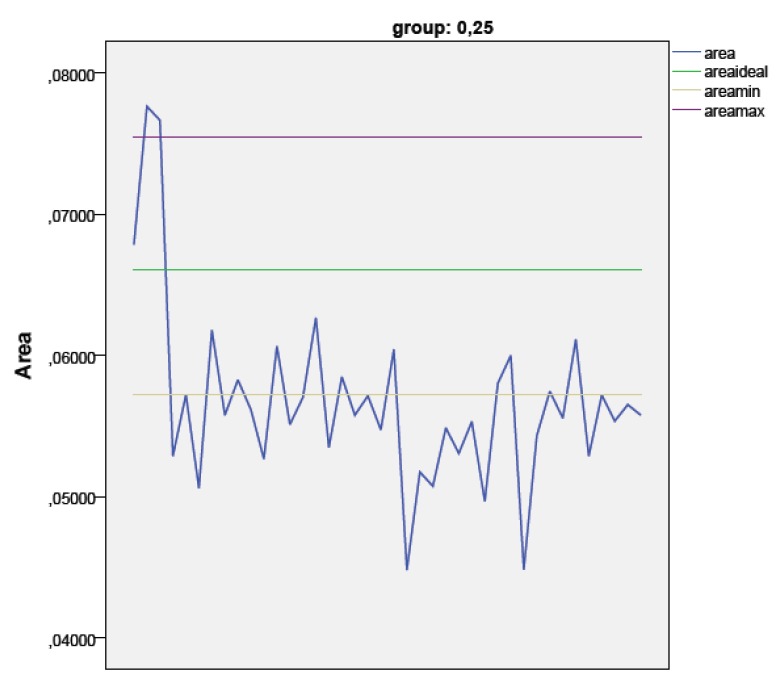
Graphic 1 - The blue line shows the Area values distribution in group 1. The green line shows the ideal area value, while purple and yellow line define the acceptable tolerance of metal (2%).

**Fig. (3):**
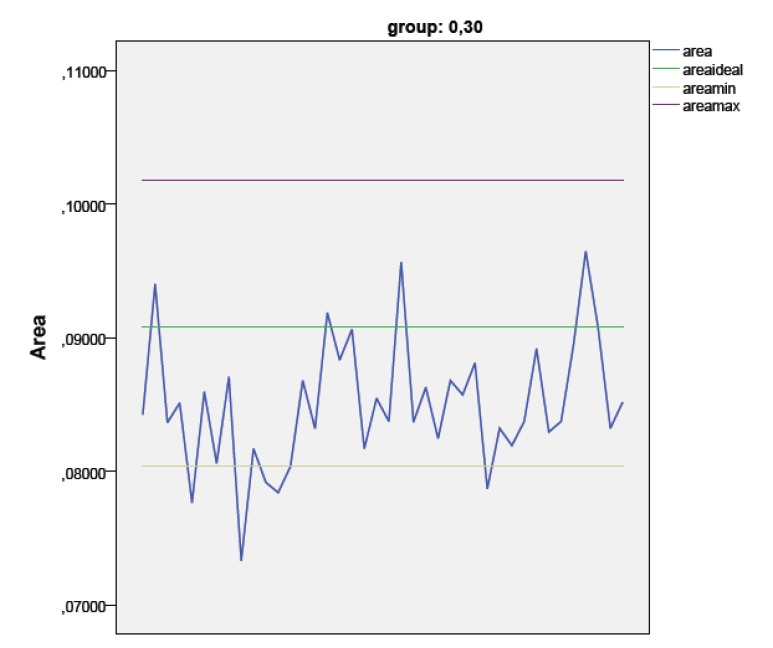
Graphic 2 - The blue line shows the Area values distribution in group 2. The green line shows the ideal area value, while purple and yellow line define the acceptable tolerance of metal (2%).

**Fig. (4):**
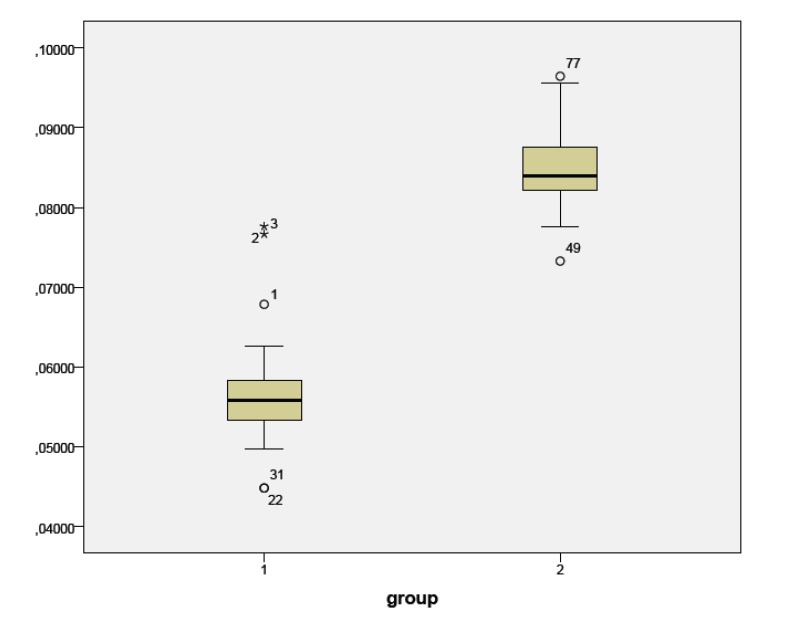
Graphic 3 – the box-and-whisker plot shows the distribution of area values in both 0.25 and 0.30 group. Whiskers represent 1.5 I QR while outliers are shown as circles and stars.

The diameter (assuming an ideal circular shape) of most far from the media samples in the group 1 and 2 has been calculated, the maximum diameter in group 1 was 0.32 mm and the minimum 0.24 mm; in group 2 the maximum diameter was 0.34 mm and the minimum 0.30 mm (Figs. **2**-**4**).

## 4 DISCUSSION

Thermafil technique is an extremely simple and fast technique, gold standard in curved and long canals, it is the only technique with apex closed by thermo plasticized gutta-percha for sure [8, 16].

Thermafil technique showed significantly higher percentage of gutta-percha-filled area than the lateral condensation technique at all segments of the root canals instrumented with hand files [9-17]. This finding corroborates with the findings of Samadi and De-Deus [18, 19] who reported the same results for Thermafil group in comparison with the lateral condensation in the cross-sectional root canal areas instrumented with hand files.

Among the various indications in literature about the apical control of gutta-percha with the Thermafil technique, the most reported is to cut 1mm of gutta-percha that protrudes from the thermafil carrier to reduce its apical excesses [14, 20-22]. There is no scientific study that demonstrates this cut really avoids overfillings. We have scientific evidence instead that the apical control of plastic carriers affects the filling quality.

Based on the results highlighted in this study, a new protocol of carrier selection and management for root canal filling is proposed by Vittoria *et al*.

**Fig. (5):**
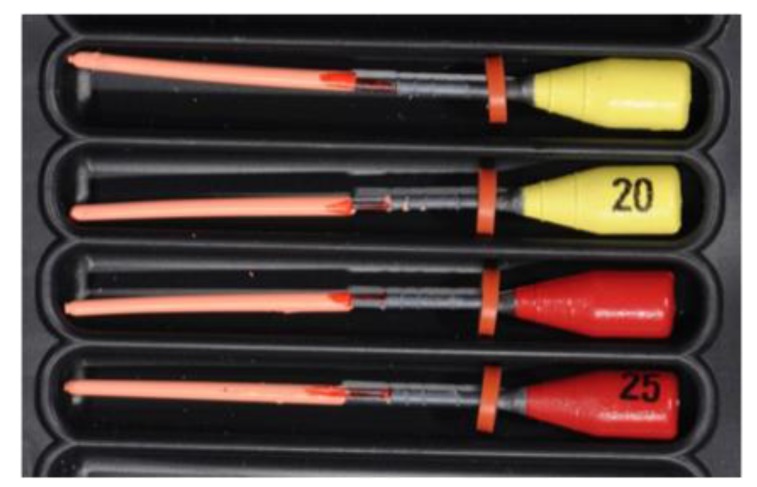
Sterile dermatological marker to create a reference between plastic core and gutta-percha sleeve.

Due to the longitudinal slot on the carrier, it is essential to have a mark which allows to find the correct position of the gutta-percha compared to the carrier at the time of covering (Fig. **5**). If this position is not found, the gutta-percha breaks down when it is reaffixed to the carrier. Such marking could be obtained with sterile dermatological marker or with a blade incision. Then the Thermafil corresponding to the diameter measured at LL (or 1 mm from the LL) is selected and gently cleaved from the gutta-percha, using two fingers with sterile gloves. Any gutta-percha residue on the carrier must be carefully removed to prevent it from dropping and blocking the canal during the test.

The carrier is tested at 1 mm from the working length with pressure; if the carrier should slide more apically it would be controlled and modified by a scalpel, shortening it over the LL +1 mm. The so modified carrier must reach 1mm from the working length without being able to go even further if pushed into the canal,we also suggest an x-ray control.

By using the marker as a guide, gutta-percha is gently repositioned on the carrier, and then the excess of gutta is removed with a scalpel until we have 1 mm or 1.5 mm of gutta exceeding the shortened carrier.

A thermafil obturator was then created, individualized to the canal that has to be filled. The tested carrier in the canal is the same that will close it removing the risk of dimensional variations and cross-infection.

The proposed modified technique allows to accurately determine the position and diameter of the carrier within the canal because it has been tested inside it, so the technique can be controlled also with large apex, removing the risk of cross-infection, it gives us the possibility to adapt a thermafil blister to different sizes, it removes the dimensional variables that could be encountered with classical technique, making apical control more predictable.

The plastic core is the only part of the Thermafil system that the clinician can control as the guttaperca became fluid. We've found in literature [14] a relation between apical position of the plastic core and sealing ability of the system. We've also found a lack in literature of measurement of these plastic cores. Our results showed that a consideration about that in clinical protocols should be done.

The disadvantage is for large apical diameters where carrier could be too shortened not reaching the working length anymore.

The authors of this study therefore propose separate blisters with the only plastic carriers, or better in cross-linked gutta-percha, to be able to try in the canal to choose that one that best suits and possibly modify it, and blisters with gutta-percha that could be attached to the selected carrier.

## CONCLUSION

The mean value of the diameters in Group 1 and 3 (Thermafil 25) was 0.27 mm in group 2 and 4 (Thermafil 30) the mean value was 0.33 mm.

Within the limitations of this study, from a clinical point of view, and regarding only this sample, in the most unfortunate hypothesis, for thermafil 25 if we select the smallest carrier we measured as verifier (0,24 mm) and we obture canal with the bigger one (0,32 mm) we will have a dimensional variable of 8% and a certain canal subtraction, in the case of Thermafil 30 the maximum possible variation found is 4%.

We can also conclude that a single heating process does not affect clinically the plastic carrier dimensions.
